# Multiple intracardiac masses: myxoma, thrombus or metastasis: a case report

**DOI:** 10.1186/s13256-015-0650-4

**Published:** 2015-08-26

**Authors:** Wei-Chieh Lee, Min-Ping Huang, Morgan Fu

**Affiliations:** 1 Division of Cardiology, Department of Internal Medicine, Kaohsiung Chang Gung Memorial Hospital, Chang Gung University. College of Medicine, 123, Ta Pei Road, Kaohsiung City, 83301 Taiwan; 2 Division of General Medicine, Department of Internal Medicine, Kaohsiung Chang Gung Memorial Hospital, Chang Gung University, College of Medicine, 123, Ta Pei Road, Kaohsiung City, 83301 Taiwan

**Keywords:** Intracardiac mass, Right atrium myxoma, Thrombus

## Abstract

**Introduction:**

The incidence of multiple intracardiac mass is rare. The differential diagnosis of intracavitary mass lesions includes benign, malignant primary, secondary metastatic cardiac tumors, or thrombus.

**Case presentation:**

We report the case of a 49-year-old Asian woman, who experienced a 2-week history of progressive exertional dyspnea, orthopnea, bilateral lower limb edema and palpitations. Transthoracic echocardiography showed one fixed round hyperechoic mass with central necrosis over the left ventricular apex, one oscillating hyperechoic nodule over the anterior mitral annulus and one irregularly heterogeneous mass bulging out from the lateral wall of the right atrium. The incidence of multiple myxomas is rare. Unfortunately, high tumor marker, serum lactic dehydrogenase and serum uric acid levels were also present. We could not differentiate between diagnoses of multiple myxomas with thrombi or multiple metastatic tumors.

**Conclusions:**

Primary intracardiac tumors are rare. Approximately 75% are benign, and approximately 50% are myxomas, which have an incidence of 0.0017% in the general population. Multiple intracardiac myxomas account for less than 5% of all cases of myxoma. Our case was an atypical picture of right atrial (RA) myxoma, as it was located in the RA lateral wall and extended to the RA auricle at the junction among the superior and inferior vena cava. Two masses in the left ventricle (LV) were thrombi and resolved after heparinization. Initially, elevated tumor markers and high serum uric acid and high serum lactic dehydrogenase levels were related to necrotic tumor-derived tissue, decompensated heart failure with pleural effusion and renal insufficiency. We share our experience of multiple intracardiac masses. Whether the intracardiac mass is benign or malignant, we recommend surgery due to the possibilities of systemic or pulmonary massive embolism, infection, arrhythmia and sudden death if the thrombus ruptures or the mass dislodges.

**Electronic supplementary material:**

The online version of this article (doi:10.1186/s13256-015-0650-4) contains supplementary material, which is available to authorized users.

## Introduction

The incidence of multiple intracavitary masses is rare. Echocardiography is a good tool to detect intracardiac masses, and the differential diagnosis must include thrombus, vegetation and a foreign body. Multiple intracardiac myxomas account for less than 5% of all cases of multiple intracardiac masses [1]. Secondary or metastatic tumors are 20–40 times more frequent than primary tumors. We share our experience about the approach to multiple intracardiac masses and show evidence of intracardiac thrombus resolution after treatment.

## Case presentation

**Fig. 1:**
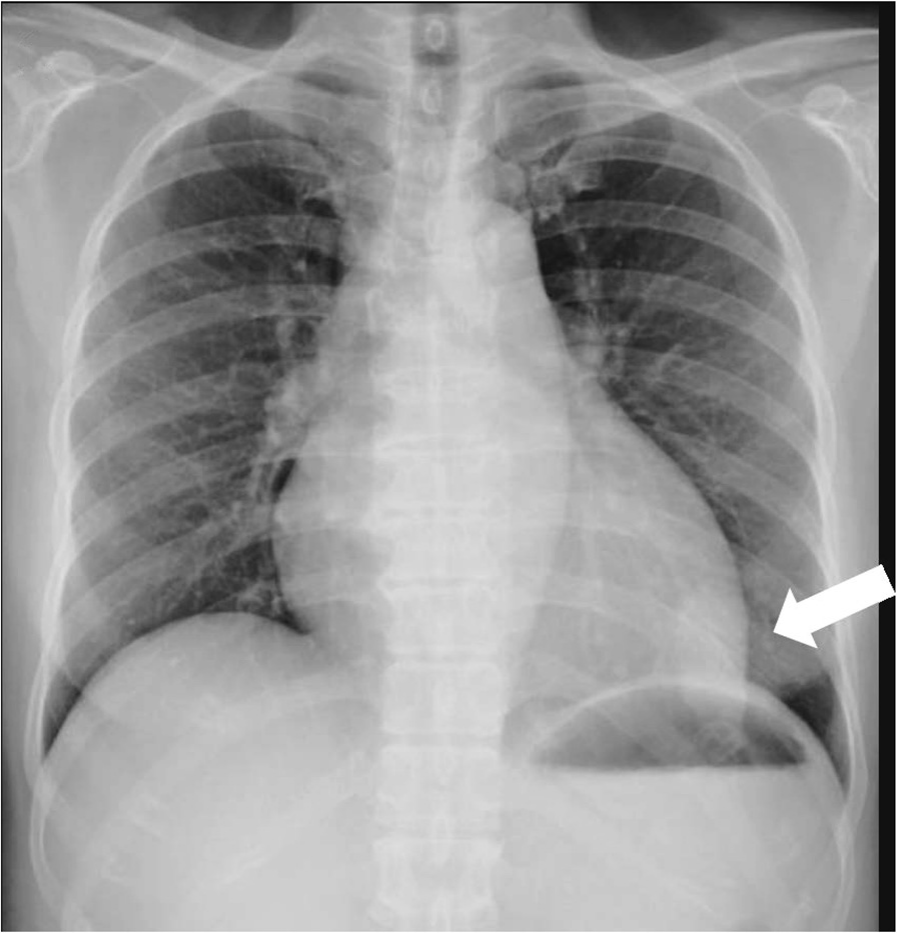
Lower left lung patch (*white arrow*) and bilateral lung infiltration

**Fig. 2:**
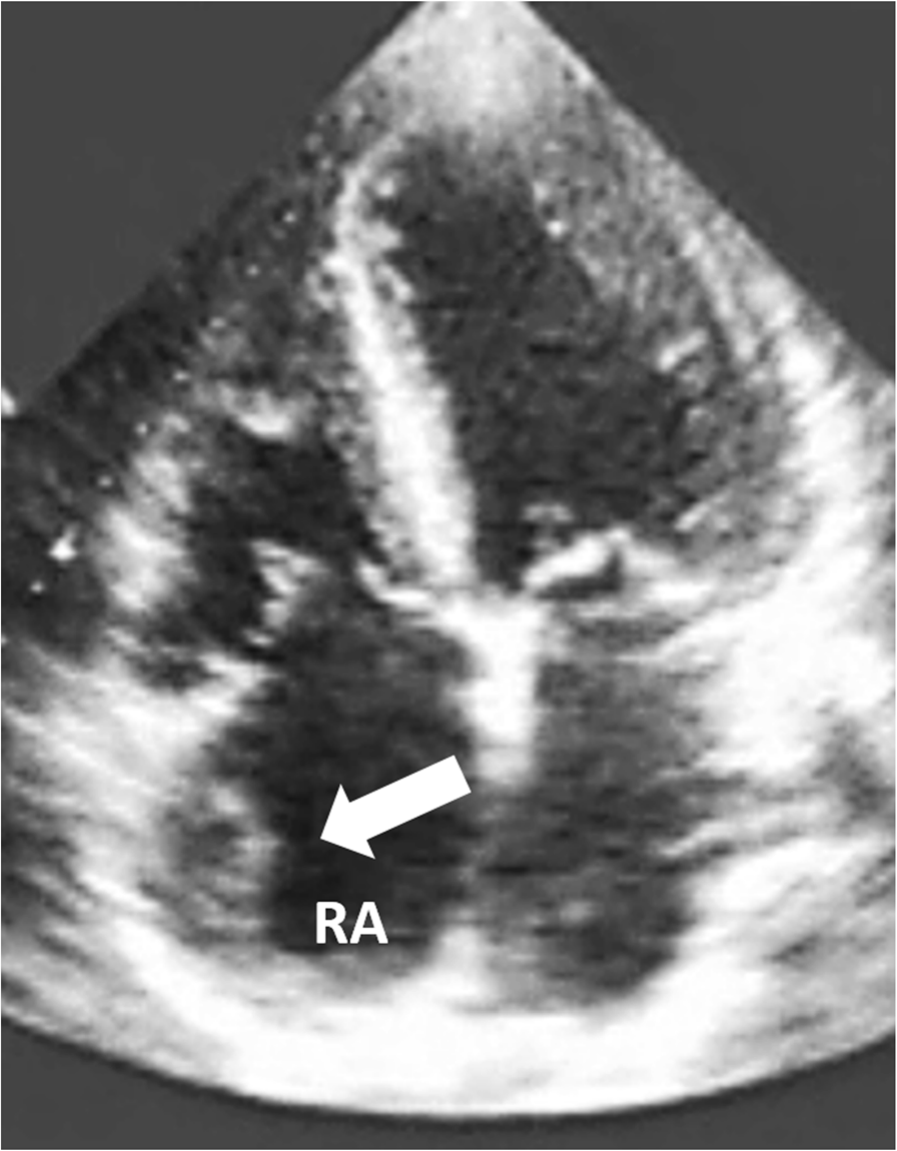
On transthoracic echocardiogram, one irregular and heterogeneous mass (5.6cm^2^) over the lateral wall of the right atrium (RA) (*white arrow*)

**Fig. 3:**
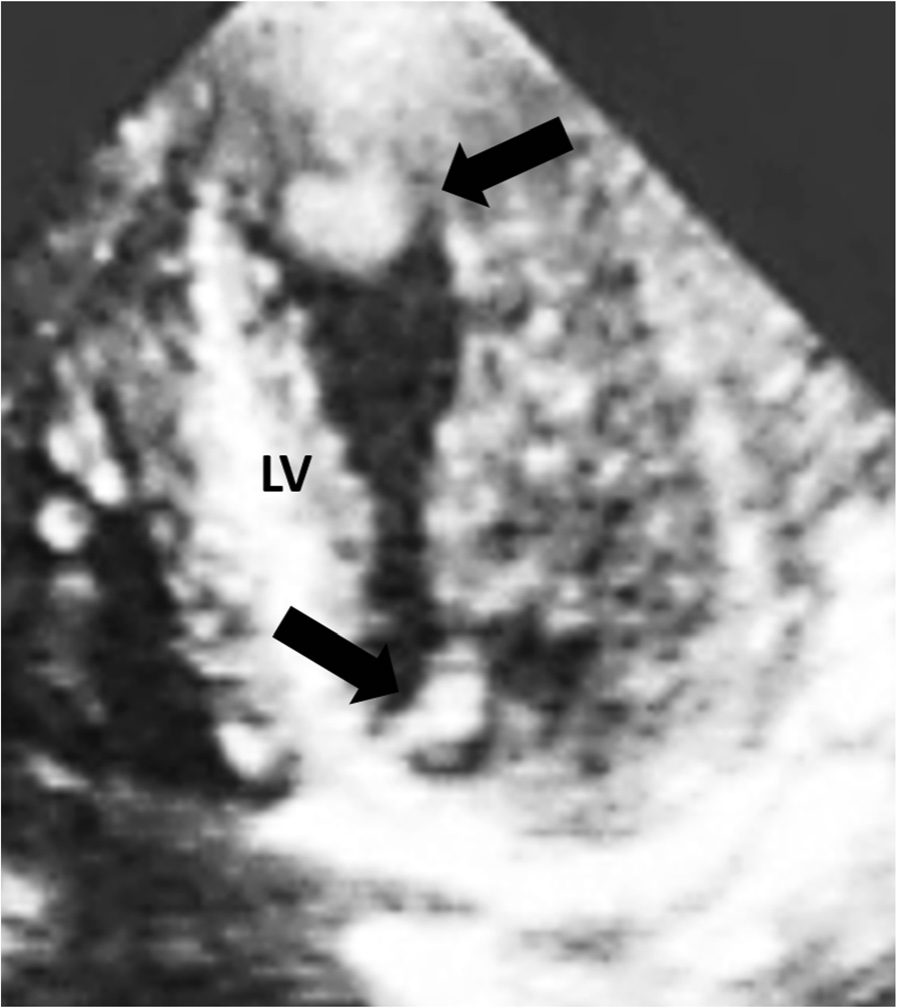
One fixed round hyperechoic mass with central necrosis over the left ventricular apex (*upper black arrow*), and one oscillating hyperechoic nodule over the anterior mitral annulus (*lower black arrow*). LV: left ventricle

A 49-year-old Asian woman without systemic disease was admitted to our hospital with a 2-week history of progressive exertional dyspnea, orthopnea, bilateral lower limb edema and palpitation. Previously, she had been in good health except for mildly elevated blood pressure. A physical examination revealed a blood pressure of 195/138mmHg and a rapid pulse rate of 163 beats/minute. Jugular venous distention to 18 cm was noted, a chest examination revealed bilateral lower lung crackles, and a cardiac examination showed an irregularly rapid beat without an audible murmur. No palpable mass was detected in her neck, axillary or inguinal areas. Her lower limbs showed grade II edema. An electrocardiogram showed atrial flutter. Chest radiography showed a lower left lung patch and bilateral lung infiltration (Fig. 1). A hemogram showed leukocytosis and a high D-dimer (14.14mg/L). Biochemistry test results revealed renal insufficiency (creatinine 3.11mg/dL) and high serum lactic dehydrogenase (LDH) (1367U/L) and uric acid (20.9mg/dL) levels. Mildly elevated troponin-I (0.067ng/mL), creatine kinase-MB (CK-MB) (11.9ng/mL and B-natriuretic peptide (BNP) (704pg/mL) were also present. Transthoracic echocardiography (TTE) showed one fixed round hyperechoic mass with central necrosis over the left ventricular apex, one oscillating hyperechoic nodule over the anterior mitral annulus and one irregular and heterogeneous mass bulging out from the lateral wall of the right atrium (RA) (Figs. 2 and 3; Additional file 1: Video S1).

**Fig. 4:**
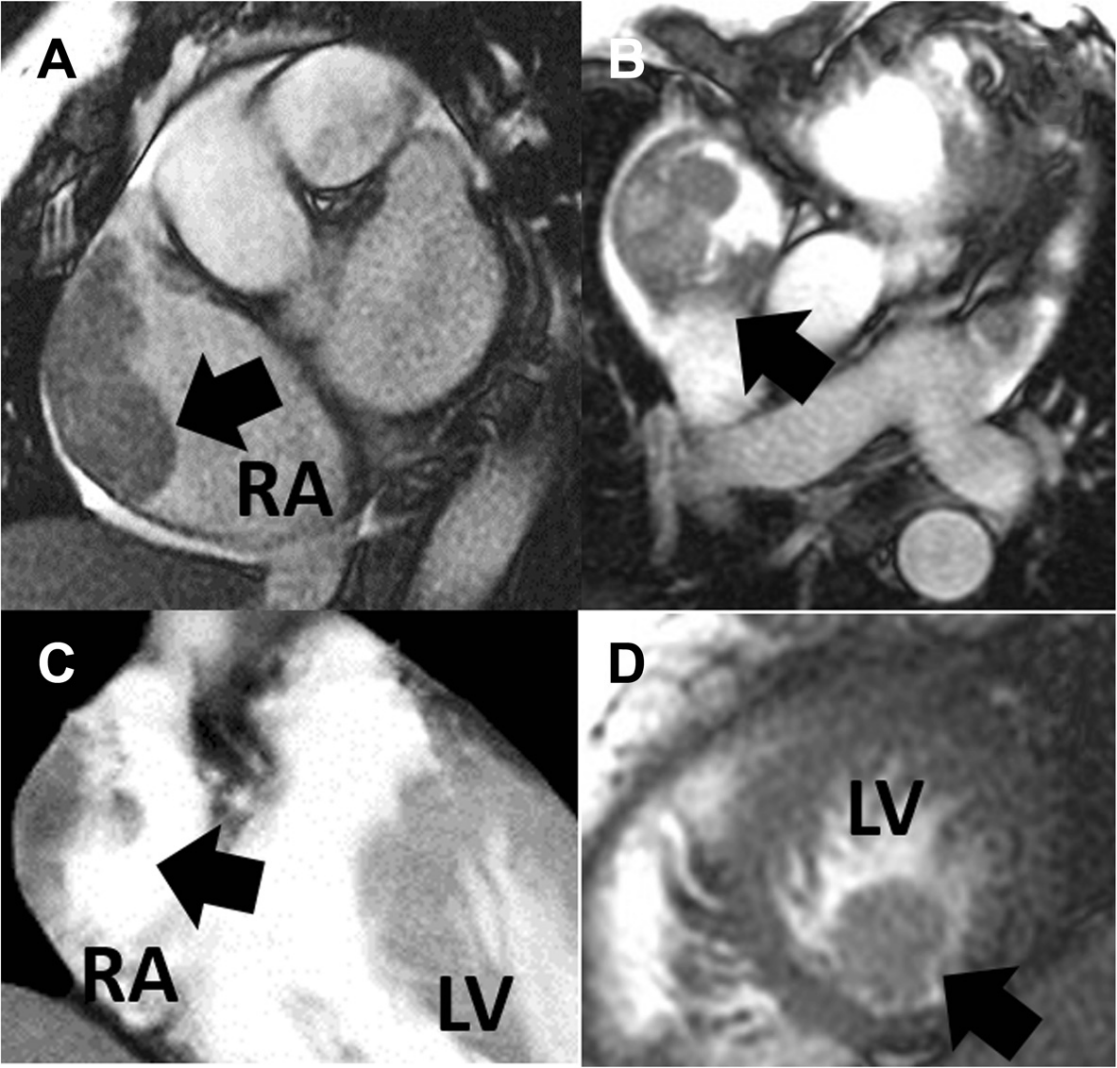
On cardiac magnetic resonance imaging (MRI) as cardiac-gated, cine gradient-echo “bright blood” magnetic resonance images, one 6.0 × 2.3cm lobulated mass bulging out from the lateral wall of the right atrium (RA) (**a**, **b**, **c**; *black arrows*) and another small nodule in the left ventricular apex (**d**; *black arrow*). LV: left ventricle

Multiple intracardiac masses were noted, and malignancy was suspected. Elevated tumor markers were noted, and her cancer antigen 125 (CA-125) level was 298.10U/mL, cancer antigen 199 (CA-199) was 52.00U/mL and cancer antigen 153 (CA-153) was 33.80U/mL. Cardiac magnetic resonance imaging (MRI) was arranged for differential diagnosis of multiple intracardiac masses. The image showed one 6.0 × 2.3cm lobulated mass bulging out from the lateral wall of the RA along with another two small nodules in the left ventricular apex and anterior aspect of the mitral valve. The one RA mass and two LV masses did not invade into the cardiac walls. All masses were near isointense on T1-weighted image (T1WI) and hyperintense on T2-weighted image (T2WI). We favored a multiple myxoma diagnosis (Fig. 4).

**Fig. 5:**
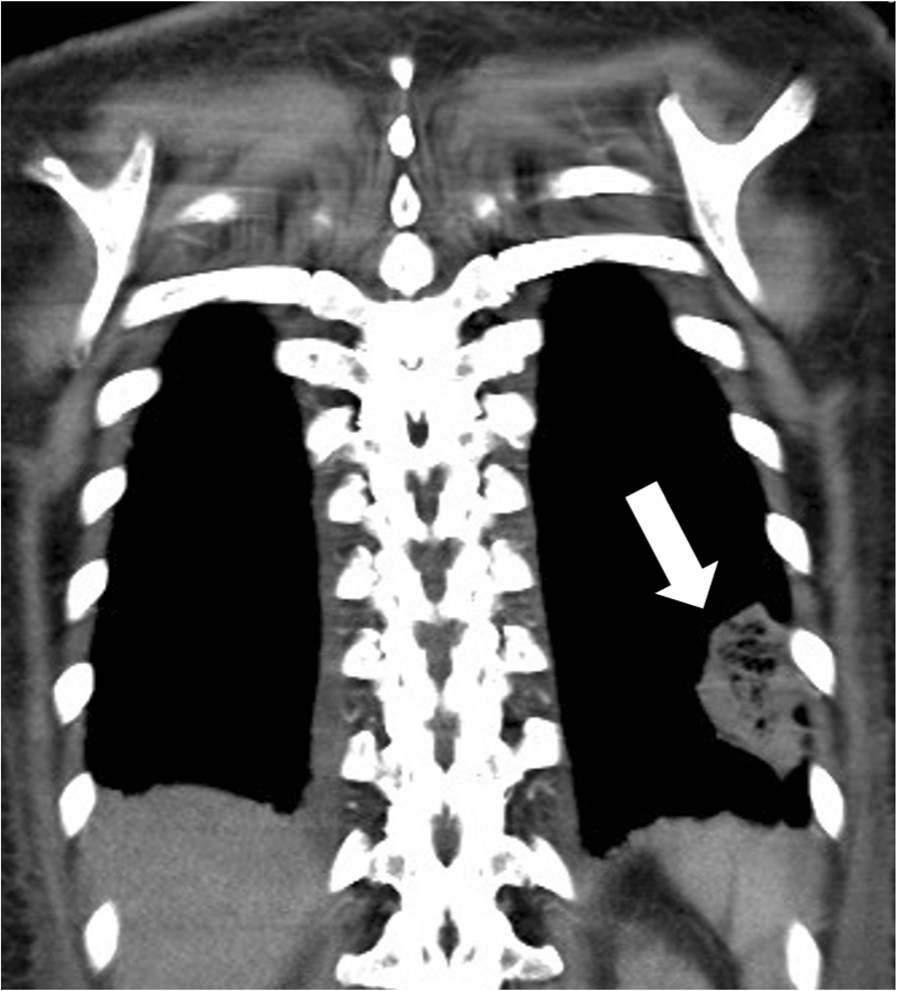
On enhanced chest computed tomography (CT), a consolidated patch with central necrosis over the lower left lung (*white arrow*)

On enhanced chest computed tomography (CT), a consolidated patch with central necrosis over the lower left lung and enlarged left anterior mediastinal and right paratracheal lymph nodes were also noted (Fig. 5). However, no definite lung nodule or mass or pulmonary embolism was detected on her chest CT scan. Despite these findings and the high tumor marker, we could not differentiate between diagnoses of multiple myxomas or multiple metastatic lung tumors.

**Fig. 6:**
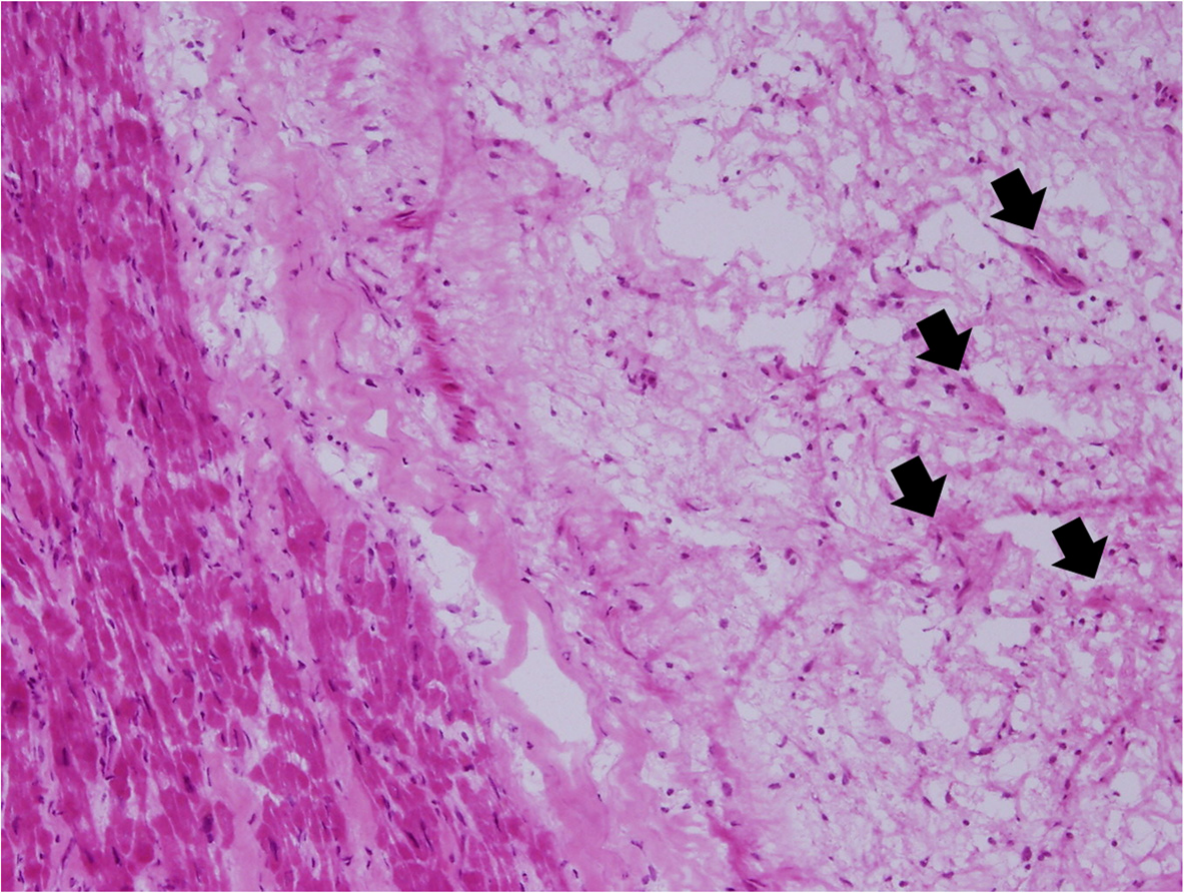
Ten times view showed that the left side was composed of muscle cells and the right side showed bland-looking oval-shaped stellate tumor cells on a fibromyxoid background (*black arrows*)

Heparinization was administered and her sinus rhythm spontaneously returned 2 days after medical treatment for heart failure and atrial flutter. Fortunately, her renal function improved and her serum LDH and uric acid levels returned to normal a few days later. We arranged a lung biopsy and echo-guided cardiac biopsies for an advanced etiologic survey. However, repeat cardiac biopsies and lung biopsies can only detect necrotic tissues or blood clots. Therefore, an open-heart biopsy and tumor excision were performed 3 weeks later and showed an RA lateral wall tumor with clots over its surfaces. The tumor extended to the RA auricle at the junction among the superior vena cava (SVC), RA and inferior vena cava (IVC). RA mass pathology with a 10 times view showed myxoma (5.1 × 3.1 × 1.5cm^3^) in which the left side was composed of muscle cells and the right side contained bland-looking, oval-shaped stellate tumor cells on a fibromyxoid background (Fig. 6). Interestingly, no mass was found in the LV, and the thrombus disappeared after heparinization.

The final diagnosis was RA myxoma and LV thrombi that resolved after heparinization. At a follow-up 1 year after surgery, our patient had returned to her daily activities, and all laboratory data, including tumor markers, had returned to normal ranges. Subsequent TTE showed adequate LV performance, and there were no further atrial flutter episodes. The consolidated patch over the lower left lung, which we favored as an arrhythmia- or myxoma-related emboli-induced lung patch, also resolved 1 month later. We suspected that initially elevated tumor markers and high uric acid and high LDH levels were related to necrotic tumor-derived tissue, decompensated heart failure with pleural effusion and renal insufficiency.

## Discussion

The incidence of multiple intracavitary masses is rare. The differential diagnosis of intracavitary mass lesions includes benign, malignant primary, secondary metastatic cardiac tumors and thrombus. Because an intracardiac lesion can be detected by echocardiography, the differential diagnosis must include thrombus, vegetation and a foreign body. However, intracardiac metastases, even though rare, should also be included in the differential diagnosis, as well as infectious and nonbacterial thrombotic or marantic endocarditis [2].

Cardiac tumors represent 0.2% of all tumors found in humans. These tumors are divided into primary or secondary/metastatic. Secondary or metastatic tumors are 20–40 times more frequent than primary tumors. Primary intracardiac tumors are rare. Approximately 75% are benign, and approximately 50% are myxomas, which have an incidence of 0.0017% in the general population [3]. Atrial myxomas are the most common (approximately 20–30%) primary intracardiac tumor in adults. Two-thirds of myxomas arise in the left atrium, whereas others arise in the RA, ventricles, SVC or pulmonary veins [4]. Only 15–20% of cardiac myxomas originate from the RA [5]. Multiple intracardiac masses are rare, and multiple intracardiac myxomas account for less than 5% of all cases [1]. The typical myxoma morphology is generally polypoid, often pedunculated, rarely sessile and round or oval, with a smooth or gently lobulated surface as observed by echocardiography [6]. RA myxomas usually originate in the fossa ovalis or the base of the interatrial septum [7]. Our case was an atypical picture of RA myxoma, as it was located in the RA lateral wall and extended to the RA auricle at the junction among the SVC and IVC. Two masses in the LV were thrombi and resolved after heparinization. The lower left lung consolidation was a suspected pulmonary thromboembolism, and also resolved after anticoagulation therapy. High levels of tumor markers, LDH and uric acid indicated a possible diagnosis of secondary malignancy.

Imaging modalities such as CT and MRI can provide additional information when echocardiography cannot delineate the extent of cardiac wall involvement. If myxoid tissue is abundantly available, the lesion will have an elevated signal in the T2WI, whereas if the fibrous component prevails, the mass will appear hypointense. Enhancement of the tumor is correlated with its histology, and the areas that are not enhanced upon imaging analysis correspond to necrosis or cystic degeneration. Enhancement is the key difference between myxomas and thrombi; the latter do not appear enhanced except in rare cases of vascularized thrombi [8].

Myxoma surfaces may have smooth or lobulated macroscopic features. Oval, rounded, and irregular shapes have been described, and a brownish color appears to predominate. The consistency of myxomas also varies from firm to gelatinous. The microscopic features of myxomas are characterized by a myxoid matrix rich in mucopolysaccharides in which polygonal cells with an eosinophilic cytoplasm can be detected. The polygonal cells may appear as star- or nest-shaped and may be multinucleated. Microscopic characteristics including mitoses, necrosis, or pleomorphisms are usually not detected or are eventually present as mild findings [3].

The classic triad found in patients with cardiac myxoma is characterized by obstruction of blood flow, constitutional symptoms and thromboembolic events. The obstruction of blood flow leads to intermittent heart failure, and, similar to systemic nonspecific flu-like malaise symptoms, there is usually a low fever of long duration, arthralgia, anorexia, and thromboembolic events. RA myxoma in particular can obstruct the tricuspid valve, causing signs and symptoms of right-sided heart failure, peripheral edema, ascites, hepatic congestion, and syncope. The signs and symptoms caused by myxomas are atypical and highly variable and can result in a difficult diagnosis of this neoplasia. According to the size, mobility and location of the tumor as well as physical activity and body position, patients may have an asymptomatic course or progress with thromboembolic events that may even lead to sudden death [9, 10]. We share our experiences regarding RA myxomas with LV thrombi and show evidence of thrombi resolution by heparinization. Whether the intracardiac mass is benign or malignant, we still recommend surgery due to the possibilities of systemic or pulmonary massive embolism, infection, arrhythmia and sudden death if a thrombus ruptures or the mass dislodges.

## Conclusions

We share our experiences regarding an atypical picture of RA myxoma, which was located in the RA lateral wall with extension to the RA auricle at the junction among the SVC, RA and IVC. Initially, clinical presentation was arrhythmia as atrial flutter and decompensated heart failure. This myxoma was also accompanied by two LV thrombi and pulmonary thromboembolism. Although high tumor markers, LDH and uric acid levels suggested a possibly secondary malignancy diagnosis. The most common mass was thrombus. In our case, the LV thrombi and pulmonary thromboembolism were resolved by heparinization. Whether the intracardiac mass is benign or malignant, we recommend surgery due to the possibilities of systemic or pulmonary massive embolism, infection, arrhythmia and sudden death if the thrombus ruptures or the mass dislodges.

## Consent

Written informed consent was obtained from the patient for publication of this case report and any accompanying images. A copy of the written consent is available for review by the Editor-in-Chief of this journal.

## Additional file

Additional file 1: **Video S1.**

## References

[1] Peachell JL, John C, Bentley MJ, Taylor GA. Biatrial myxoma: a rare cardiac tumor. Ann Thorac Surg. 1998;65:1768–9. 10.1016/s0003-4975(98)00206-9 9647099

[2] Reynen K, Köckeritz U, Strasser RH. Metastases to the heart. Ann Oncol. 2004;15:375–81. 10.1093/annonc/mdh086 14998838

[3] Nina VJ, Silva NA, Gaspar SF, Raposo TL, Ferreira EC, Nina RV, et al. Atypical size and location of a right atrial myxoma: a case report. J Med Case Rep. 2012;6:26. 10.1186/1752-1947-6-26 PMC3277469 22269461

[4] Lamparter S, Moosdorf R, Maisch B. Giant left atrial mass in an asymptomatic patient. Heart J. 2004;90:24–6. 10.1136/hrt.2003.028910 PMC1768196 15084572

[5] Diaz A, Di Salvo C, Lawrence D, Hayward M. Left atrial and right ventricular myxoma: an uncommon presentation of a rare tumour. Interact Cardiovasc Thorac Surg. 2011;12:622–3. 10.1510/icvts.2010.255661 21273256

[6] Reynen K. Cardiac myxomas. N Engl J Med. 1995;333:1610–17. 10.1056/NEJM199512143332407 7477198

[7] Stolf NA, Benício A, Moreira LF, Rossi E. Right atrium myxoma originating from the inferior vena cava: an unusual location with therapeutic and diagnostic implications. Rev Bras Cir Cardiovasc. 2000;15:255–8.

[8] Motwani M, Kidambi A, Herzog BA, Uddin A, Greenwood JP, Plein S. MR imaging of cardiac tumors and masses: a review of methods and clinical applications. Radiology. 2013;268:26–43. 10.1148/radiol.13121239 23793590

[9] Vale Mde P, Freire Sobrinho A, Sales MV, Teixeira MM, Cabral KC. Giant myxoma in the left atrium: case report. Rev Bras Cir Cardiovasc. 2008;23:276–8. 10.1590/s0102-76382008000200020 18820794

[10] Oliveira R, Branco L, Galrinho A, Abreu A, Abreu J, Fiarresga A, et al. Cardiac myxoma: a 13-year experience in echocardiographic diagnosis. Rev Port Cardiol. 2010;29:1087–100. 21066964

